# Supplementary figures and images for: Emerging Clostridioides difficile ribotypes have divergent metabolic phenotypes
Source: mSystems. 2025 Feb 27;10(3):e01075-24. doi: 10.1128/msystems.01075-24 (PMC11915817; doi:10.1128/msystems.01075-24)

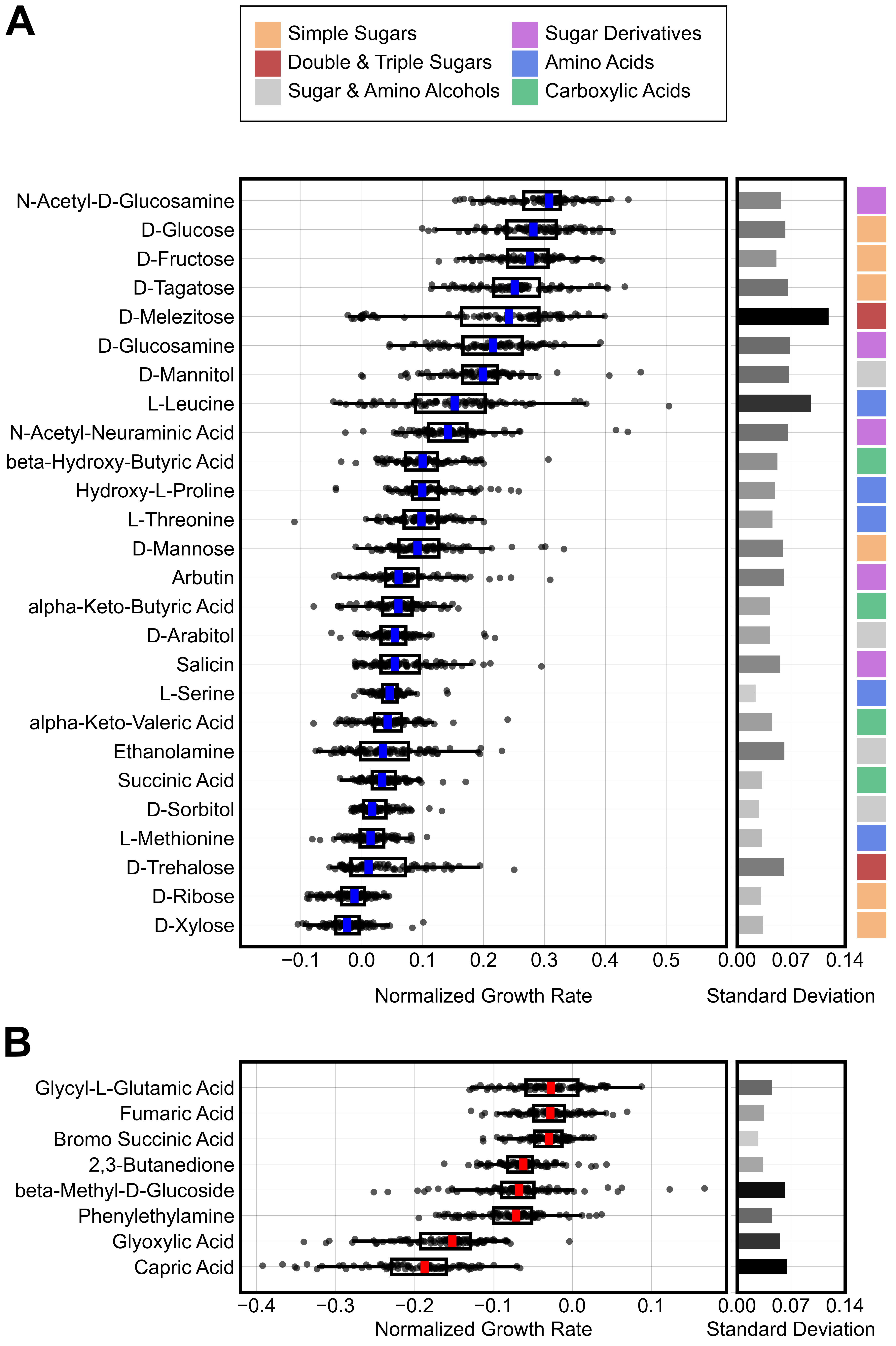

Supplement: Figure S1 — Distribution of normalized growth rates by substrate. [file msystems.01075-24-s0001.tiff]

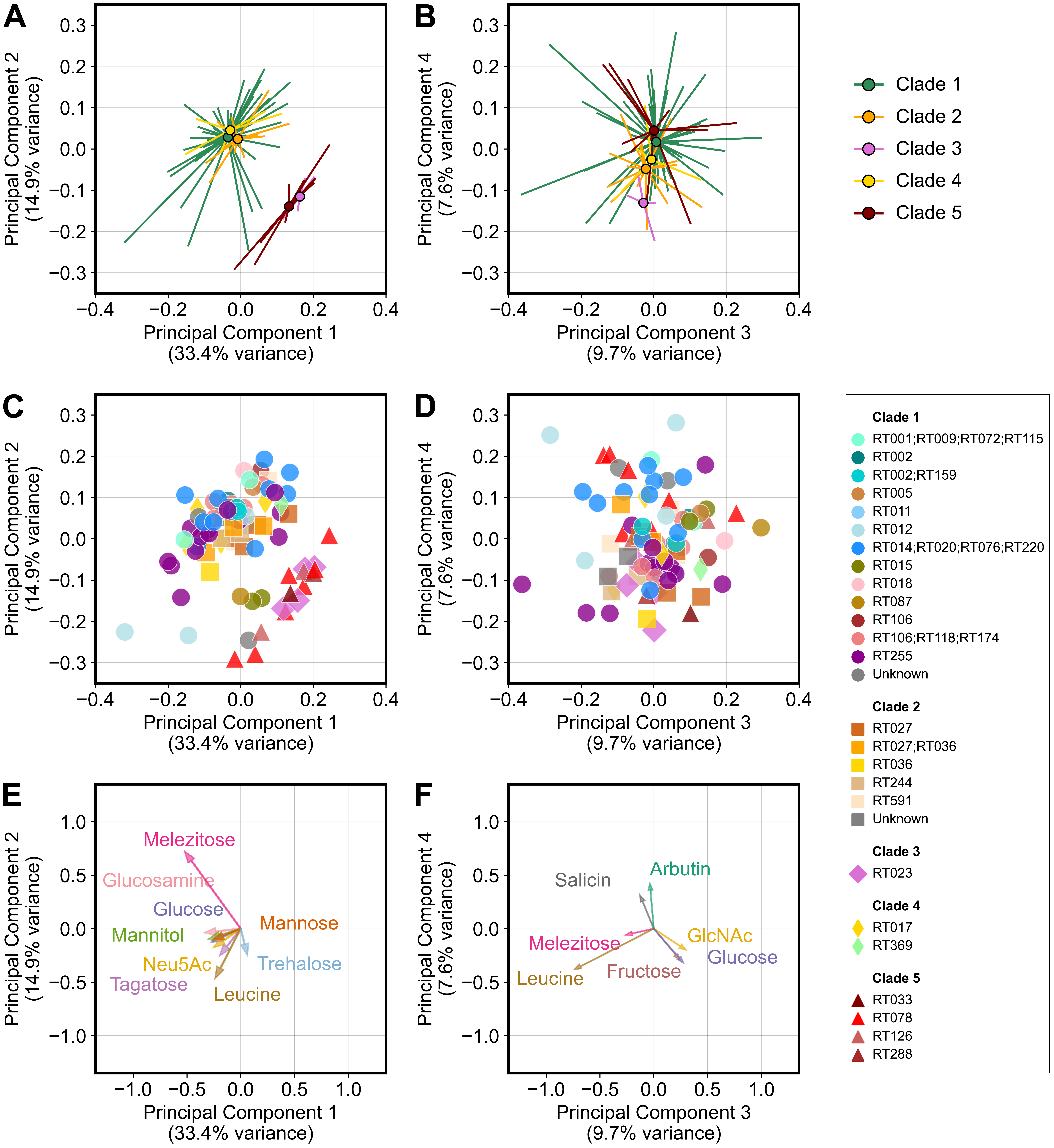

Supplement: Figure S2 — Principal component analysis of normalized growth rates. [file msystems.01075-24-s0002.tiff]

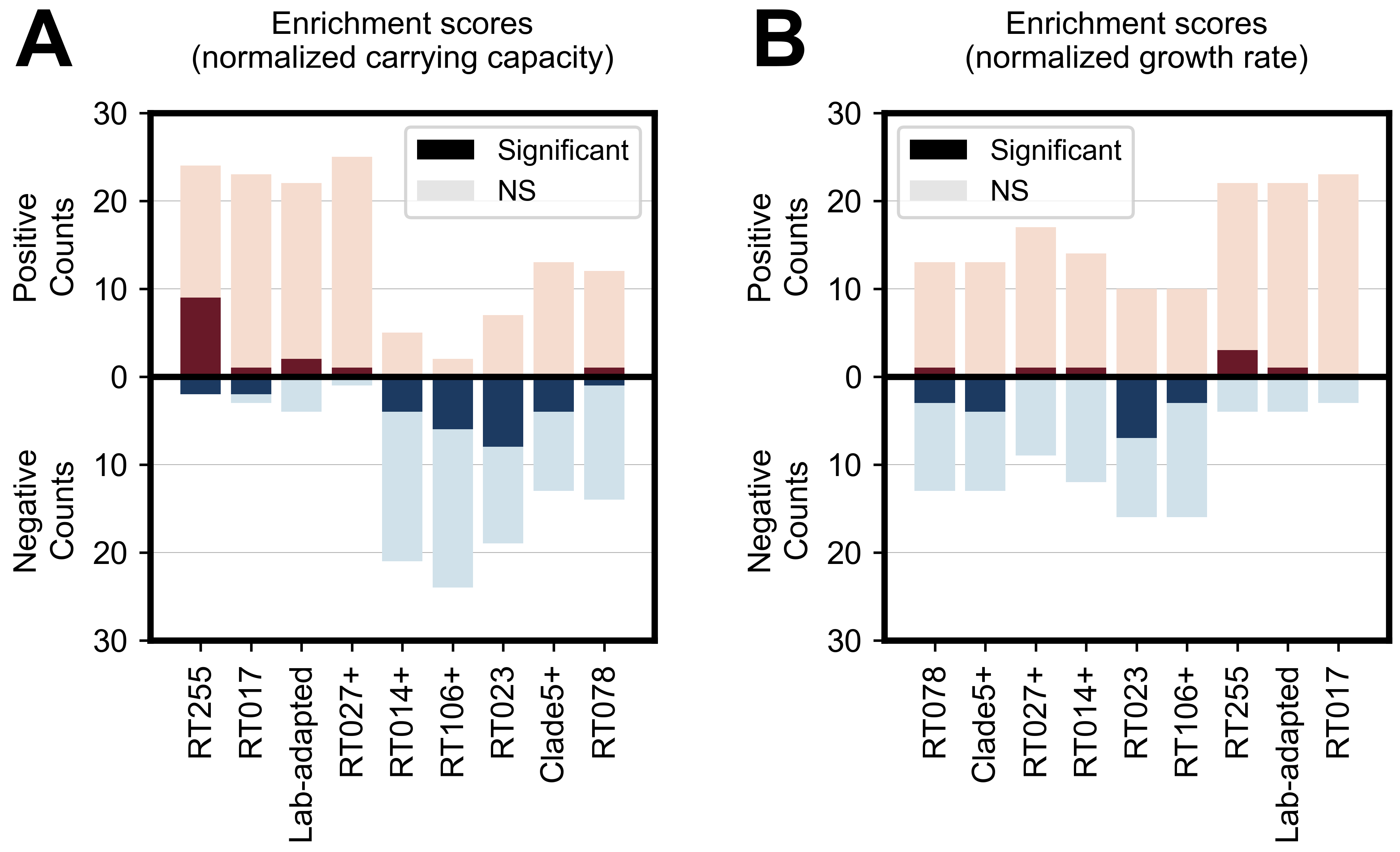

Supplement: Figure S4 — Summary of strain set enrichment analysis. [file msystems.01075-24-s0004.tiff]

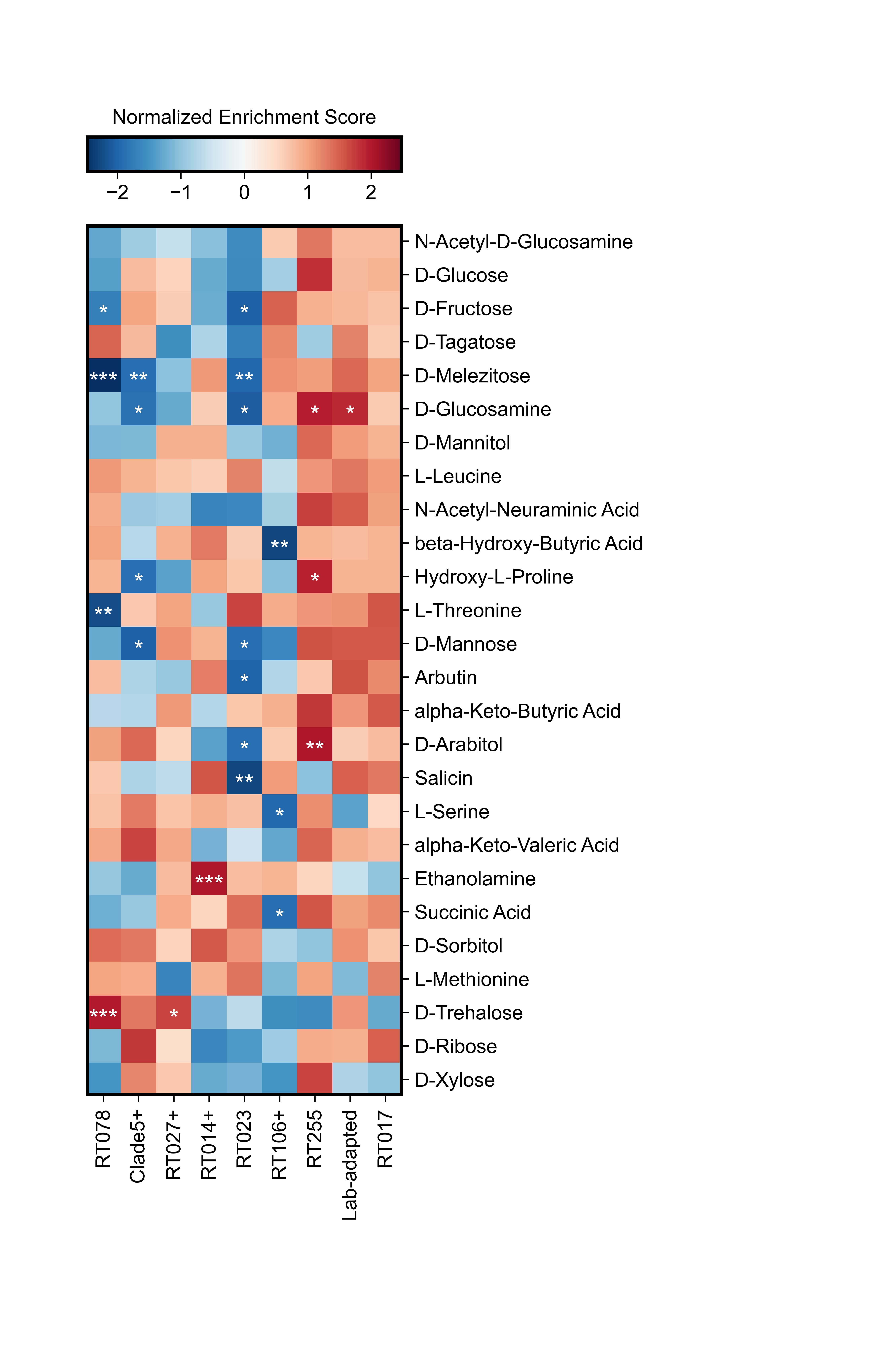

Supplement: Figure S5 — Strain set enrichment analysis of normalized growth rates. [file msystems.01075-24-s0005.tiff]

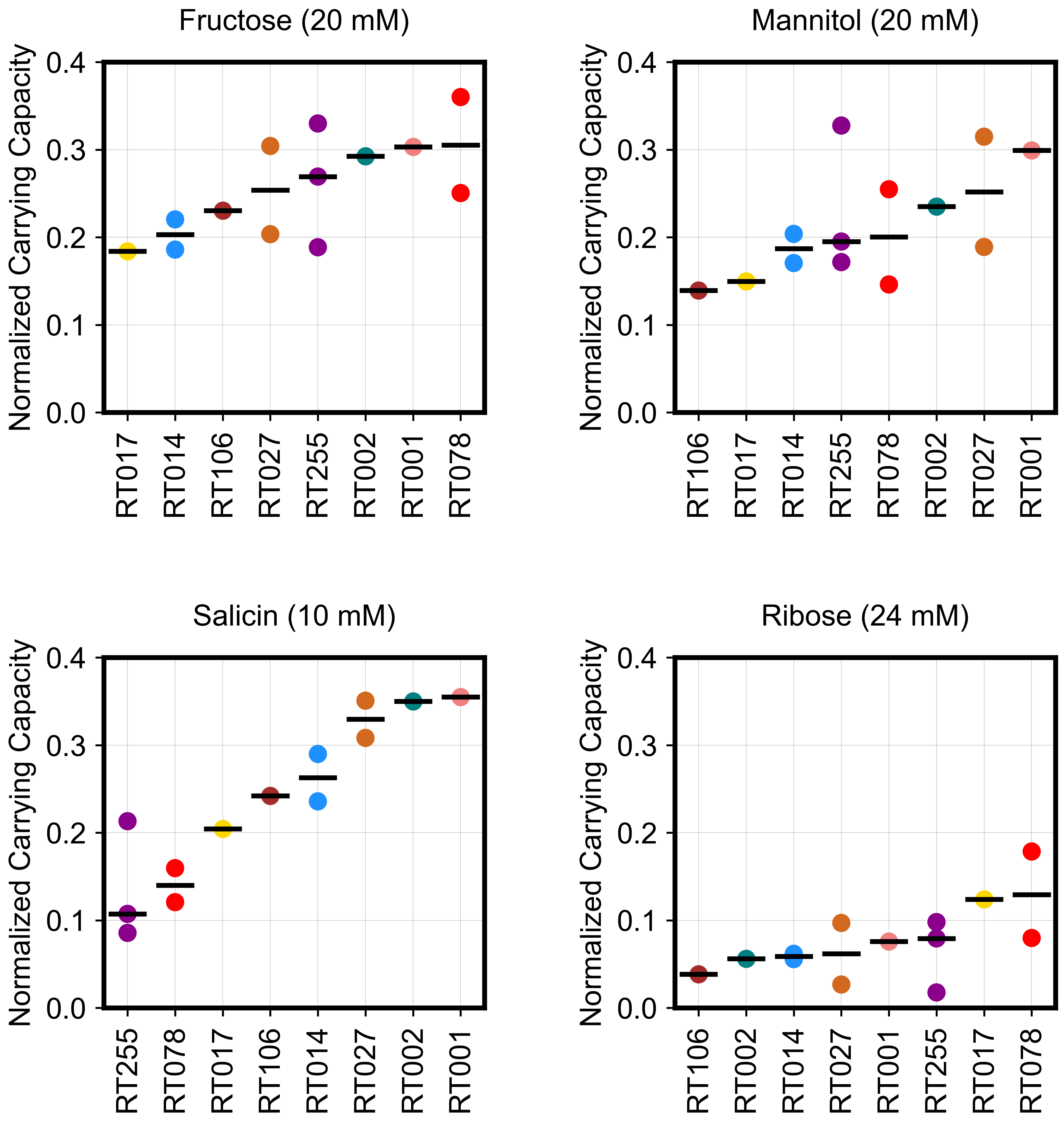

Supplement: Figure S6 — Growth validation experiment on four substrates. [file msystems.01075-24-s0006.tiff]

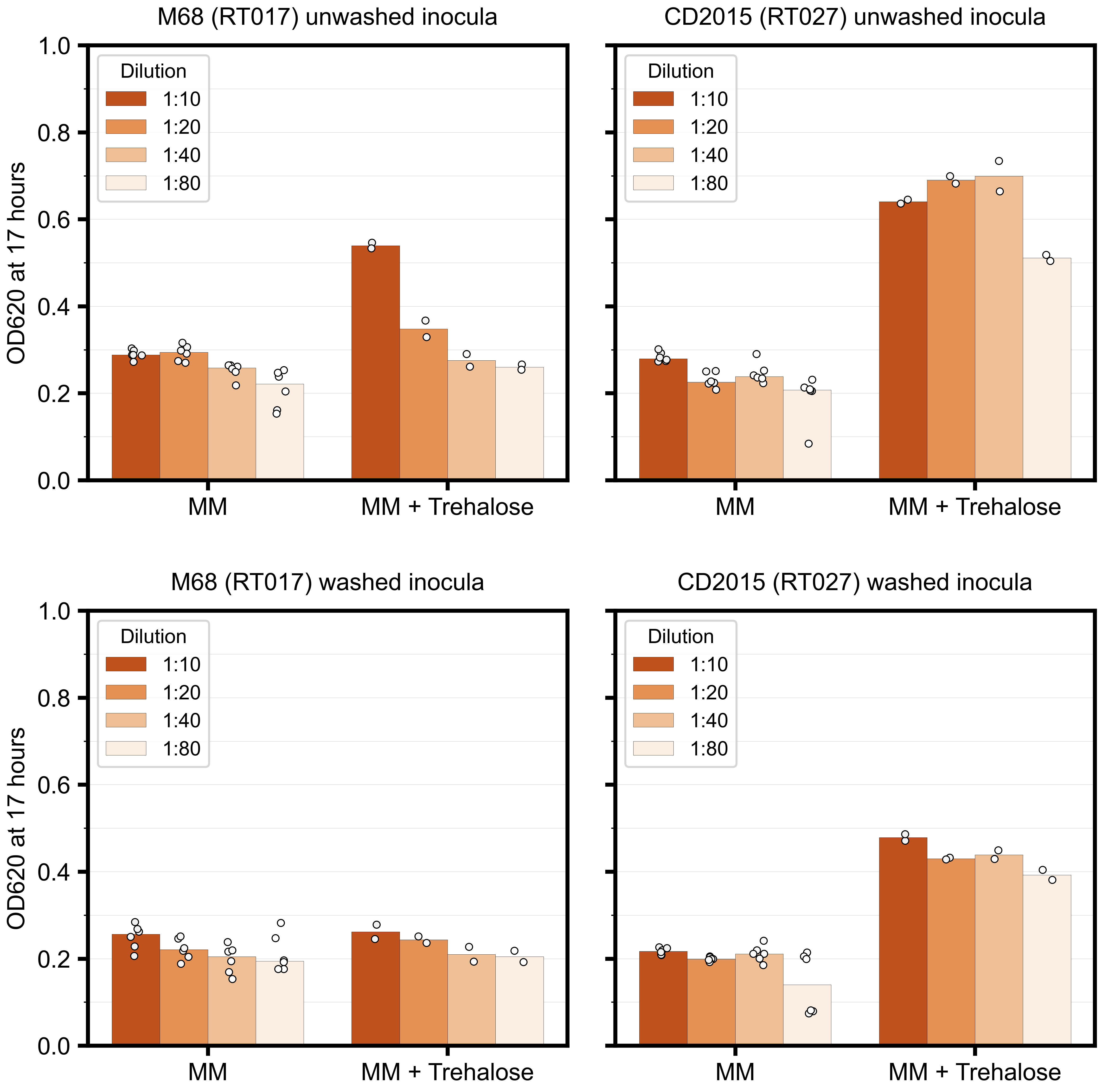

Supplement: Figure S7 — Experiment for limiting factor in yeast extract. [file msystems.01075-24-s0007.tiff]
