# Supplementary material for: Emerging Clostridioides difficile ribotypes have divergent metabolic phenotypes
Source: mSystems. 2025 Feb 27;10(3):e01075-24. doi: 10.1128/msystems.01075-24 (PMC11915817; doi:10.1128/msystems.01075-24)
Supplement: Supplemental legends — Legends for supplemental figures and tables. [file msystems.01075-24-s0008.docx]

**SUPPLEMENTAL FIGURE LEGENDS**

**Figure S1.** **Distribution of normalized growth rates for *C. difficile* isolates on the top 26 substrates.** This figure complements Figure 1 which displays the distribution of normalized carrying capacities. Normalized growth rate is defined as the difference in the exponential growth rate on minimal media supplemented with a single carbon source versus on minimal media only. (A) Top and (B) bottom panels display normalized growth rates for carbon sources that supported and inhibited total growth, respectively, as shown in Figure 1. Box plots display the median and interquartile range of values. Whiskers extend to the farthest point within 1.5x of the interquartile range. Horizontal bars on the right side display the standard deviation for each distribution and the shading of these bars scales with the standard deviation value.

**Figure S2.** **Principal component analysis of the normalized growth rates for *C. difficile* isolates on the top 26 substrates.** This figure complements Figure 2 which visualizes the principal component analysis based on the normalized carrying capacities. (**A**-**B**) Top panels display the principal component analysis of the normalized growth rates of isolates on the top 26 carbon substrates. Isolates are grouped by clades with circles indicating the centroid for each group and lines pointing to the location of each isolate in the ordination plots. (**C**-**D**) Middle panels display the same principal component analysis and further delineate the ribotype of each isolate with colors and clade with shapes, as indicated in the legend on the right. (**E-F**) Bottom panels visualize how certain substrates contribute to the position of isolates on the principal components (loading factors). Left column displays analysis for the first and second principal components while the right column displays analysis for the third and fourth principal components.

**Figure S3. Normalized carrying capacity (K) for all isolates on the top carbon sources.** Colors and markers indicate the ribotype and clade of each isolate as shown in Figure 2. Whiskers extend to the farthest point within 1.5x of the interquartile range.

**Figure S4. Summary chart for the strain set enrichment analysis shown in Figures 3 and S5.** Bars display the total count of positive enrichments (red bars) and negative enrichments (blue bars) for each ribotype based on strain enrichment analysis using (**A**) normalized carrying capacity or (**B**) normalized growth rate. Dark and light shading indicates enrichments that are statistically significant or not significant (NS), respectively. Isolate sets were ordered on the x-axis to match the order in Figures 3 and S5 respectively.

**Figure S5. Heatmap of enrichment scores based on normalized growth rates for C*. difficile* isolates on the top 26 substrates.** This figure complements Figure 3 which displays the enrichment scores based on the normalized carrying capacities. Heatmap displays which ribotypes are positively or negatively enriched for growth rate on each of the top carbon sources. Normalized enrichment scores were computed using strain set enrichment analysis. Strain groups (columns) are hierarchically clustered based on similarity of their normalized enrichment scores, while substrates (rows) are ordered from top to bottom based on the median growth of all isolates as shown in Figure S1. For each substrate, statistical significance was estimated with a permutation-based test procedure and corrected with the Benjamini-Hochberg method. **P* < 0.05, ***P* < 0.01, ****P* < 0.001.

**Figure S6. Growth validation experiment recapitulated patterns detected by the Biolog phenotype microarray assays.** *C. difficile* harvested during exponential growth was inoculated into minimal media with one of four carbon substrates. Colors indicate the ribotype of each isolate as shown in Figure 2.

**Figure S7. Unknown limiting factor in yeast extract enabled ribotype 017 to grow on trehalose.** Ribotype 017 isolate M68 and ribotype 027 isolate CD2015 were grown overnight in brain heart infusion supplemented with high yeast extract, serially diluted in defined minimal media, then grown on minimal media (MM) alone or minimal media supplemented with 40 mM trehalose. Overnight cultures were either washed in defined minimal media, or not, prior to dilution and inoculation. Left and right panels display results for M68 and CD2015 respectively. Top and bottom panels display results for growth using unwashed and washed inocula respectively. Using unwashed inocula, the maximum optical density of M68 on trehalose decreased with increasing dilution factors which suggests that a limiting factor is necessary for growth on trehalose. In addition, M68 was unable to grow on trehalose after washing likely because washing eliminated this limiting factor.

**SUPPLEMENTAL TABLE LEGENDS**

**Table S1. Sources of *C. difficile* isolates.**

**Table S2. Molecular typing of *C. difficile* isolates.**

**Table S3. Composition of *C. difficile* minimal media.**
